# Green Tea Extract Enhances the Oxidative Stability of DHA-Rich Oil

**DOI:** 10.3390/antiox10060982

**Published:** 2021-06-19

**Authors:** Caroline Waingeh Nain, Gwennaëlle Berdal, Phan Thi Phuong Thao, Eric Mignolet, Marine Buchet, Melissa Page, Yvan Larondelle

**Affiliations:** 1 Louvain Institute of Biomolecular Science and Technology, UCLouvain, Croix du Sud, 4-5, L7.07.03, B-1348 Louvain-la-Neuve, Belgium; caroline.nain@uclouvain.be (C.W.N.); gwennaelle.berdal@student.uclouvain.be (G.B.); eric.mignolet@uclouvain.be (E.M.); marine.buchet@uclouvain.be (M.B.); melissa.page@uclouvain.be (M.P.); 2 Faculty of Food Science and Technology (FST), Vietnam National University of Agriculture (VNUA), Hanoi 131000, Vietnam; phanphuongthao@vnua.edu.vn

**Keywords:** oxidation, DHA-rich oil, natural antioxidants, alpha-tocopherol, catechins, green tea extract (GTE)

## Abstract

Docosahexaenoic acid (DHA) is one of the most important omega-3 polyunsaturated fatty acids, with proven health-promoting properties. However, oils with a very high content in DHA (DHAO) are extremely susceptible to oxidation, which affects shelf stability and limits incorporation in food products. Green tea extracts (GTE) are potential candidates for the protection of these oils, but their use in such oils has not been previously reported. This study investigated the effect of GTE (160 ppm, 400 ppm, 1000 ppm) and α-tocopherol (80 ppm, 200 ppm, 500 ppm) on the oxidative stability of a DHAO over a 9-week storage at 30 °C. The oxidative status was monitored during storage by the measurement of peroxide value (PV) and *p*-anisidine value (*p*-AV). Changes in eicosapentaenoic acid (EPA) and DHA content, as well as in catechins and tocopherol contents, were also evaluated. The addition of GTE enhanced the oxidative stability of DHAO by reducing the formation of peroxides and secondary oxidation products, whereas α-tocopherol had no significant effect on the PV of oil during storage but led to a significantly higher *p*-AV. The EPA and DHA content of DHAO was stable in GTE-supplemented samples whereas a decrease was observed in the control and α-tocopherol-supplemented samples. GTE also delayed the degradation of tocopherols initially present in the oil, while catechins resulting from the addition of GTE decreased progressively during the storage period.

## 1. Introduction

Docosahexaenoic acid (DHA, C22:6n-3) is a long chain omega-3 fatty acid and an essential component of a healthy diet. Its consumption has been associated with a decrease in the risk of cardiovascular and inflammatory diseases, cancer and neurological disorders, among others [1,2]. DHA can be produced endogenously from alpha-linolenic acid (C18:3n-3) by humans but the rate of biosynthesis is low and insufficient to meet physiological demands [3]. Dietary sources are therefore required to meet the recommended daily intake of 250 mg DHA/day set for adults by the French Food Safety Agency (AFSSA) [4]. The traditional sources of long-chain omega-3 fatty acids, namely DHA and eicosapentaenoic acid (EPA, C20:5n-3) in the diet are oily fish and seafoods, which are naturally rich in these fatty acids. Microalgae are a primary source of DHA in the marine ecosystem and in recent years, the cultivation of microalgae for oil production has emerged as a new source of DHA. DHA-rich oils (DHAO), being highly unsaturated, are especially susceptible to oxidation and require special handling to prevent the development of off-flavours [5].

Lipid oxidation is one of the causes of quality deterioration in oils and food. When unsaturated lipids are exposed to metal ions, heat or light, free radicals are formed, which in turn react with oxygen to form hydroperoxides [6]. Hydroperoxides are very unstable and further break down into aldehydes, ketones and alcohols, which are responsible for the off flavours in oxidized oils [7,8]. The rate of oxidation is positively correlated to the number of double bonds in the fatty acids used as building blocks for the lipids of a food item. Thus, oils with high polyunsaturated fatty acid (PUFA) ratios tend to oxidize easily [9]. It is therefore essential to protect these oils from oxidation in order to improve product shelf life and stability. Incorporating antioxidants is one of the effective methods to achieve this protection.

Antioxidants are compounds that inhibit lipid oxidation by either scavenging free radicals, chelating transition metals, quenching singlet oxygen and/or inhibiting the activity of photoactivated sensitizers [10]. Synthetic antioxidants such as butylated hydroxytoluene (BHT), butylated hydroxyanisole (BHA) and tert-butylhydroquinone (TBHQ) are added to oils to delay oxidation but, because of the health risk associated with these compounds, their usage is regulated in most countries [11]. Hence, there is an increasing interest in natural antioxidants, especially with the surge in consumers’ preference for natural over synthetic products [12].

Most natural antioxidants are derived from plant materials such as fruits, vegetables, herbs and spices [11,13]. These antioxidants from plants are mainly polyphenols, carotenoids and vitamins [14]. Alpha-tocopherol, which is the major tocopherol in many edible oils such as almond, peanut, olive, wheat germ, cottonseed and sunflower oils, has been effective in delaying oxidation in some oils, although it acts as a pro-oxidant if used in high concentrations [15,16,17]. The optimal effective level of α-tocopherol varies from one oil to another with optimal efficiency reported at 50 ppm for sunflower oil [18], 100 ppm for olive oil [19] and 250–500 ppm for corn oil emulsions [20]. Other natural antioxidants such as quercetin and boldine, as well as a mixture of supercritical extracts of rosemary and α-tocopherol, were shown to be effective in improving the oxidative stability of oils with high contents of omega-3 fatty acids (≥80% of total fatty acids) [21,22]. Additionally, investigated for antioxidant activity are extracts from grape seed, apple peel, tomato peels, rambutan peel and green tea leaves [23,24,25,26,27]; and some of these extracts exhibit similar or better antioxidant properties compared to synthetic antioxidants.

Green tea extracts (GTE) are produced from green tea leaves by conventional solvent extraction, ultrasound-assisted extraction, microwave-assisted extraction or supercritical fluid extraction among others [28]. The antioxidant activity of GTE is associated with its content of catechins, which prevent oxidation by chelating metal ions or by donating hydrogen atoms to free radicals, thereby blocking or slowing down the free radical chain reaction [29]. The major catechins of green tea include epigallocatechin (EGC), epicatechin (EC), epigallocatechin gallate (EGCG) and epicatechin gallate (ECG) [30]. The antioxidant activity of GTE has been demonstrated in various food systems including baked products [23,31], meat and pork [32], beverages [33], table spreads [34], as well as bulk oils, including marine oils [35,36]. To our knowledge, however, the effect of GTE has never been evaluated on hypersensitive oils with an extremely high content in DHA.

This study investigates the effectiveness of GTE as a natural antioxidant in an oil with a very high DHA content. The antioxidant effect of GTE was compared to that of α-tocopherol, which were both incorporated into DHA-rich oil samples and tested for oxidation at 30 °C. Peroxide and para-anisidine values were monitored during the oxidation process. We also measured changes in DHA and EPA levels, as well as catechin and tocopherol contents. The results indicate that GTE is an efficient alternative for the stabilization of DHA-rich oils. Owing to the importance of dietary DHA for health, the present study offers a new tool to allow for its inclusion in prevention programs against various diseases.

## 2. Materials and Methods

### 2.1. Materials

DHA-rich oil (DHAO) made from deep-sea fish oil concentrate was kindly provided by Atrium innovations Inc. (Antwerpen, Belgium). Green tea leaf extract (GTE) containing 20% catechin was a product of Naturex (Avignon, France); D-α-tocopherol (1000 IU/g) was purchased from Sigma-Aldrich (Schnelldorf, Germany). All other chemicals used in this study were either of GC-grade, HPLC-grade or analytical grade.

### 2.2. Sample Preparation for Oxidative Stability Test

The effect of GTE and α-tocopherol on the oxidative stability of DHAO was evaluated using the oven storage test method for accelerated aging of oils [37]. Oil samples were prepared with added antioxidant or without antioxidant (control) according to the method of Douny et al. [38], with some modifications. In short, different concentrations of GTE (1000, 400 and 160 ppm) and of α-tocopherol (500, 200 and 80 ppm) were incorporated into DHAO. The GTE and α-tocopherol were each dissolved in absolute ethanol, vortexed for 1 min and sonicated (70 W, 40 kHz) for 15 min. A limited amount of ethanol was used so as not to exceed 4% weight of the final oil mixture. The oil and antioxidant blends were vigorously mixed with a glass rod for 3 min and flushed with nitrogen for 10 min to evaporate the ethanol. The oil samples were then distributed (10 g) in amber bottles (30 mL, Ø 2.7 cm), and the uncapped bottles were stored in the dark at 30 °C for 9 weeks. Bottles were removed from the oven after 3 weeks, 6 weeks and 9 weeks of storage and analysed for the oxidative status, fatty acid content, catechin and tocopherols. Separate sample bottles were used for each sampling time. Samples for initial analysis (0 week) were not put in the oven. The oxidative status determined by peroxide value (PV) and *p*-anisidine value (*p*-AV) was evaluated on the sampling day and the remaining oil was flushed with argon and stored at −20 °C for subsequent analyses of the other parameters. The oil samples supplemented with GTE at 1000 ppm, 400 ppm and 160 ppm were referred to as GTE1000, GTE400 and GTE160, respectively, whereas those supplemented with α-tocopherol at 500 ppm, 200 ppm and 80 ppm were referred to as Toco500, Toco200 and Toco80, respectively. All samples were prepared in triplicate.

### 2.3. Assessment of the Oxidative Status

#### 2.3.1. Peroxide Value (PV)

The PV, which measures primary oxidation products in oil samples, was determined according to the AOAC official method 965.33 [39] with some modifications. An oil sample (2.5 g) was dissolved in 15 mL of acetic acid–chloroform solution (3:2 *v*/*v*). Then, 0.25 mL of saturated potassium iodide (KI) solution was added to the mixture, shaken for 1 min, kept in the dark for 5 min and 15 mL of distilled water were added. Titration was carried out with 0.1 N sodium thiosulfate solution until the yellow colour almost disappeared. 0.5 mL of starch solution (1% *w*/*v*) was added and titration continued until the blue colour disappeared. PV was calculated using the formula below:

(1) $$\mathrm{PV}\;\left({\mathrm{meq}\;O}_{2}/\mathrm{kg}\;\mathrm{oil}\right)=\frac{\left(V\;-{\;V}_{b}\right)\;\times \;N\;\times \;1000}{m},$$

where V and V_b_ are volumes (mL) of sodium thiosulfate for sample and blank, respectively, N is the normality of sodium thiosulfate and m is the mass of oil (g).

#### 2.3.2. Para-Anisidine Value (*p*-AV)

Secondary oxidation products in oil samples were measured as *p*-AV according to the AOCS official method Cd 18–19 [40]. An oil sample of 0.3–0.5 g (g m) was dissolved in 25 mL of isooctane and absorbance was measured at 350 nm (A_b_) using a quartz cuvette. One mL of *p*-anisidine solution (0.25% in glacial acetic acid (*w*/*v*)) was added to 5 mL of this mixture and after 10 min in the dark, the absorbance was measured at 350 nm (A_s_). A blank was prepared with isooctane and measured the same way as the samples. The following equation was used to calculate *p*-AV:

(2) $$p-\mathrm{AV}\;=\frac{25\;\times \;(1{.2\;\times \;A}_{s}-{\;A}_{b})}{m},$$

where 25 is the volume of isooctane used to dissolve the oil and 1.2 is a correction factor for dilution of sample solution with 1 mL of *p*-anisidine solution.

### 2.4. Determination of Fatty Acid Composition

The method used was adapted from Mellery et al. [41]. DHAO sample (200 mg) was weighed and converted to fatty acid methyl esters (FAME) via a two-step methylation with nonadecanoic acid (C19: 0, Sigma-Aldrich) used as internal standard. First, the oil was methanolysed with 10 mL of 0.1 M KOH in methanol at 70 °C for 60 min, then cooled to room temperature. Second, an acid-catalysed methanolysis was carried out by adding 4 mL of 1.2 M HCl in methanol and incubating at 70 °C for 20 min. The FAME were then extracted by addition of hexane and Milli-Q water. The FAME were separated and quantified by running samples in a GC Trace 1310 (ThermoQuest, Milan, Italy) gas chromatograph equipped with an As Triplus autosampler (Thermo Electron, Milan, Italy), an RT2560 capillary column of 100 m × 0.25 mm internal diameter (Restek, Bellefonte, PA, USA) and a flame ionisation detector set at 255 °C. Fatty acids in the samples were identified and quantified by comparison of retention time and peak area with those of pure FAME standards (Larodan, Solna, Sweden). The data were processed using the Chromquest 5.0 data processing software (ThermoFisher Scientific, Waltham, MA, USA) and results are expressed as mg/g of oil.

### 2.5. Determination of Tocopherol Content

The alpha-, gamma- and delta- tocopherol content of oil was determined by high-performance liquid chromatography (HPLC). To prepare samples for analyses, 2.5 g of oil were dissolved in 25 mL of HPLC-grade hexane. The tocopherol standards (Sigma-Aldrich, St. Louis, MO, USA) were also prepared at different concentrations (1, 2, 5, 10, 20, 50 and 100 ppm) in hexane. The samples and standards were filtered through 0.45 µm syringe filters into HPLC injection vials. The tocopherols were separated and quantified by injecting 50 µL of diluted standards and samples in a chromatograph equipped with an autosampler AS3000 (Oven temperature = 35 °C, tray temperature = 15 °C), a P1000 spectra system pump (Thermo separation products, San Jose, CA, USA), a Luna 5 µm normal phase column of 250 mm × 4.6 mm internal diameter (Phenomenex, Torrance, CA, USA) and an FP-2020 fluorescence detector (Jasco, Tokyo, Japan). The mobile phase used was 2% (*v*/*v*) tetrahydrofuran in n-heptane at a flow rate of 1.8 mL/min. The data were processed using the Chromquest 5.0 data processing software and the results are expressed as µg/g of oil.

### 2.6. Quantification of Catechins in GTE and in Oil Samples

The catechin, EGC, EC, EGCG and ECG contents were determined for GTE and oil samples. GTE (0.3 g) and absolute ethanol (12 g) were weighed in a centrifuge tube. After vortexing for 1 min, the mixture was sonicated (70 W, 40 kHz) for 15 min at room temperature and centrifuged for 5 min at 1800× *g*. The ethanolic extract obtained was filtered through a 0.45 µm syringe filter for analysis by HPLC.

The catechins were extracted from oil samples supplemented with GTE using a method adapted from Pizarro et al. [42]. The oil sample (0.5 g) was weighed into a 1.5 mL Eppendorf tube to which 1 mL of 90% ethanol was added, vortexed for 1 min and centrifuged for 5 min at 20,000× *g*. A volume of 750 µL of the ethanolic phase was transferred into another Eppendorf tube (2 mL) and after eliminating the remaining ethanolic phase, a second extraction of the remaining oil phase was carried out. After the second extraction, 750 µL of the ethanolic phase was again collected and pooled with the volume collected from the first extraction. Ethanol was evaporated in a SpeedVac concentrator Savant SPD121P (ThermoFisher Scientific, Asheville, NC, USA) and the residue was solubilised in 1 mL of 1% formic acid in water. The solution was filtered through a 0.45 µL syringe filter into an HPLC vial. The HPLC used was equipped with an AS1000 autosampler (oven at 40 °C and tray at 4 °C), a P4000 pump (Thermo Separation Products, San Jose, CA, USA), a Phenomenex kinetex 2.6µm PFP 100A column (150 mm × 4.6 mm) and a DAD UV 6000 LP diode array detector (DAD; Thermo Separation Products, San Jose, CA, USA). The mobile phase consisted of solvent A (water with 1% (*v*/*v*) formic acid) and solvent B (acetonitrile with 1% (*v*/*v*) formic acid), used for analysis according to the following elution protocol: 0 min, 3% B; 5 min, 9% B; 11 min, 10.5% B; 20 min, 25% B; 25 min, 100% B; 30 min, 100% B; 37 min, 3% B; 40 min, 3% B. The flow rate was set at 1 mL/min with a sample injection volume of 10 µL. The catechins were identified and quantified by comparing retention times and peak areas with those of pure standard compounds (Extrasynthese, Genay, France). The results are expressed in µg/g.

### 2.7. Statistical Analysis

All experiments were carried out in triplicate, and results are expressed as mean ± standard deviation. An analysis of variance (ANOVA) was carried out using JMP Pro 15.2.0 statistical package to evaluate the effect of treatments during storage and the differences among the treatments. Sample means were compared by Turkey’s HSD (honestly significant difference) test with differences considered statistically significant at *p* ≤ 0.05.

## 3. Results and Discussion

### 3.1. Characteristics of DHA-Rich Oil

The DHAO had a PUFA content of 873 mg/g corresponding to 99% of total fatty acids in the oil, with the major fatty acid being DHA (626.7 mg/g = 71.1% of total fatty acids), followed by EPA (149.3 mg/g = 16.9% of total fatty acids) and omega-3 docosapentaenoic acid (54.7 mg/g = 6.2% of total fatty acids). Other fatty acids were only present in trace amounts (Table 1). The total tocopherol content of the oil was 2174.4 µg/g. The concentrations of α-tocopherol, γ-tocopherol and δ-tocopherol were 342.2 µg/g, 1380.9 µg/g and 448.3 µg/g, respectively. The oil had a good oxidative status with a PV of 2 meq O_2_/Kg and a *p*-AV of 5.2, which complied with the PV limit of 5 meq O_2_/Kg oil and the *p*-AV limit of 20 established by the Global Organization for EPA and DHA Omega-3s (GOED) and by the Codex Alimentarius [5,43].

### 3.2. Oxidative Status of Oil during Storage

**Figure 1:**
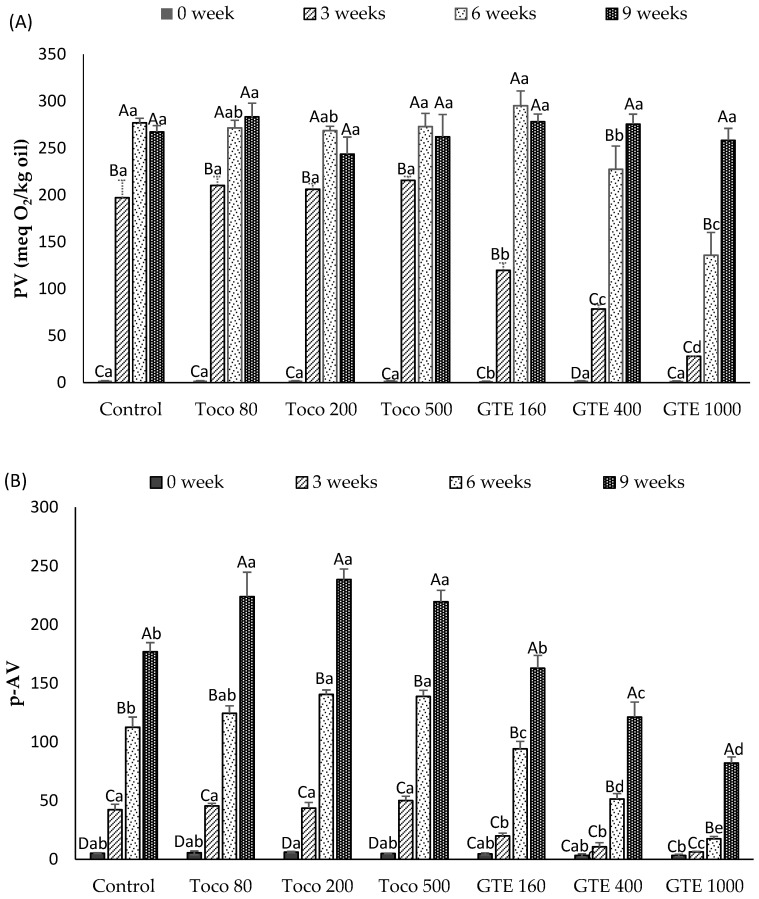
Peroxide value (PV) (**A**) and *p*-anisidine value (*p*-AV) (**B**) of DHA-rich oil (DHAO) with or without antioxidant during an oven storage test at 30 °C. Values are mean ± SD (n = 3); Different capital letters (A–D) indicate significant differences (*p* ≤ 0.05) during storage within a sample; different lower-case letters (a–e) among samples indicate a significant difference (*p* ≤ 0.05) at a specific storage time. Control = DHA-rich oil without added antioxidant; Toco80, Toco200 and Toco500 = DHA-rich oil supplemented with 80 ppm, 200 ppm and 500 ppm of α-tocopherol, respectively; GTE160, GTE400, GTE1000 = DHA-rich oil supplemented with 160 ppm, 400 ppm and 1000 ppm of green tea extract, respectively.

The changes in PV and *p*-AV of oil samples over the 9-week storage at 30 °C are presented in Figure 1. The PV of the oil samples ranged from 1.6 to 2 meq O_2_/Kg oil at the start of the storage test (0 week) and increased to different degrees in all the samples during the storage period (Figure 1A). After 3 weeks of storage, a significant increase (*p* ≤ 0.05) in PV was detected in all samples except GTE1000, in which a significant change was not detected. The PVs of control samples and tocopherol-supplemented samples increased 100-fold and were significantly higher (*p* ≤ 0.05) than values for the GTE-supplemented samples. This drastic increase in PV had been observed in fish and algae oils during accelerated storage at 40 °C, whereby PVs of up to 287.9 and 343 meq O_2_/kg were detected after 96 h of storage in some of the oils [44]. The PV of GTE1000, GTE400 and GTE160 was 85.8%, 60% and 39% less than that of the control, respectively. After 6 weeks of storage, the PV of GTE1000 samples remained significantly lower (*p* ≤ 0.05) than the PV of all other samples with a 55% lower value than the control. There were no significant differences (*p* > 0.05) observed in the PV among samples after 9 weeks of storage.

The *p*-AV of oil samples also changed during storage (Figure 1B). The *p*-AV of the control and α-tocopherol-supplemented samples significantly increased (*p* ≤ 0.05) throughout the storage period. Meanwhile, the *p*-AV of the GTE-supplemented samples were stable during the first 3 weeks of storage and remained significantly lower (*p* ≤ 0.05) than those of the other samples throughout the storage period. After 6 weeks of storage, the *p*-AV of the samples supplemented with 200 ppm and 500 ppm of tocopherol were significantly higher (*p* ≤ 0.05) than those of the control and GTE-supplemented samples, suggesting a pro-oxidant activity. The rate of increase in *p*-AV for GTE-supplemented samples was inversely related to the concentration of GTE in the samples and was in the order of GTE1000 < GTE400 < GTE160. The *p*-AV of GTE1000 was the lowest throughout the storage period and was 65% lower than the control at 9 weeks of storage. The lower increase in PV and *p*-AV of GTE-supplemented samples could be explained by the presence of catechins, which as free radical scavengers, react with free radicals formed during the primary oxidation phase, thus disrupting the oxidation process [45].

Highly unsaturated lipids, like those containing a high percentage of DHA, are especially prone to oxidation and require special handling to prevent the development of off-flavours [5]. The high oxidation rates of DHA and EPA and the instability of their hydroperoxides cause a rapid formation of secondary products. These secondary products include volatile aldehydes, ketones and other compounds, which, in turn, impart flavour reversion in fish oils [46]. The *p*-AV of GTE1000 at 6 weeks of storage, was still compliant with the *p*-AV limit of 20 established for concentrated fish oils [5,43] but surpassed this limit by week 9. Supplementing DHAO with GTE improved its oxidative stability whereas the addition of α-tocopherol increased the formation of secondary oxidation products. The effectiveness of GTE as an antioxidant in DHAO is in agreement with previous results whereby GTE at concentrations of 200 ppm, 500 ppm and 1000 ppm improved the oxidative stability of refined menhaden oil (containing 10% of DHA) and seal blubber oil (containing 7.5% of DHA) more so than α-tocopherol at 500 ppm [35]. In another study, lipid fractions of sponge-fat cakes enriched with GTE (0.02%, 0.2% and 1%) also exhibited a greater resistance to oxidation than cakes without antioxidant [23]. The effect of tocopherols in oils is strongly concentration-dependent, varying with oxidation temperature and interactions with other molecules [17,47]. Alpha-tocopherol has been shown to exert pro-oxidant effects in fish oil whereas binary or ternary antioxidant systems, containing α- or γ-tocopherol concentrates, ascorbic acid (or ascorbyl palmitate), and lecithin, were successful in improving the oxidative stability [16,48]. Similar to our results, the pro-oxidant effect of α-tocopherol (50, 100, 200 and 400 ppm) in linseed oil samples stored at 25, 40 and 60 °C for over 30 days was reported by Mohanan et al. [49], who confirmed their results in an accelerated test using the Rancimat technique. In another study, Fuster et al. [50] reported that both α- and γ-tocopherols within a concentration range of 40–200 ppm were effective in enhancing the oxidative stability of purified sunflower oil during 7 days of storage at 55 °C, while neither α- nor γ-tocopherol displayed a pro-oxidant effect at concentrations as high as 2000 ppm.

### 3.3. The Fatty Acid Composition of DHAO during Storage

The EPA and DHA contents of DHAO samples on week 0 and after 9 weeks of oxidation are presented in Table 2. There was no significant difference (*p* > 0.05) in the fatty acid composition of samples at the start of storage (week 0). The mean contents of EPA and DHA in oil samples at week 0 were in the range of 138.7–151.3 mg/g and 583.5–635.7 mg/g, respectively. After 9 weeks of storage, a significant decrease (*p* ≤ 0.05) in EPA and DHA was observed for the control, Toco80 and Toco200 samples. The addition of 500 ppm α-tocopherol also led to a visible decrease in both EPA and DHA, but this did not reach significance. In contrast, the addition of GTE had a clear protective effect on the degradation of EPA and DHA. Shi et al. [51] observed a decrease in the EPA and DHA content of spray-dried matcha-tuna oil powders stored over 12 weeks at 40 °C. An increase in the percentage of matcha (rich in catechins) in the spray-dried powders enhanced the protection of these fatty acids. In another study, the incorporation of tea catechin (1 mM) or α-tocopherol (1 mM) in an oleic and linolenic acid–rich diacylglycerol oil, had no effect on the fatty acid composition of oil during a 10-day storage at 60 °C [52]. Similar to the results observed in this study for control and α-tocopherol-supplemented samples, Phung et al. [53] reported an 8% decrease in EPA and DHA contents of hoki liver oil after 30 days of continuously bubbling the oil with oxygen gas and a 7% decrease when the oil was stored in an oven at 50 °C for 30 days.

### 3.4. Losses in Catechins

The GTE used in this study had a total catechin content of 192.4 mg/g. The major catechin was EGC (115.1 mg/g) followed by EGCG (54.1 mg/g), EC (13.5 mg/g) and ECG (8.4 mg/g). The content of catechins in samples supplemented with GTE was proportional to the quantity of GTE added, with a total content of 39.4 µg/g, 99.4 µg/g and 224.8 µg/g in GTE160, GTE400 and GTE1000 samples, respectively (Table 3). After 9 weeks of storage, the total catechin content had reduced to 19–22 µg/g, with no significant differences observed among treatments. Additionally, the percentage losses in EGC, EC and EGCG were similar within a treatment, with a loss of 54–55% for GTE160, 85–86.6% for GTE400 and 92–95% for GTE1000. The losses in ECG were lowest in each treatment, with no loss observed for GT160 samples, 15.5% for GTE400 and 43.54% for GTE1000 samples. Residual amounts of EGC, EC, EGCG or ECG among samples after 9 weeks did not differ. Based on these results, there seemed to be a minimum level beneath which the loss of catechins was no longer possible. This could explain why there was no significant difference in the PV among samples at this point.

**Figure 2:**
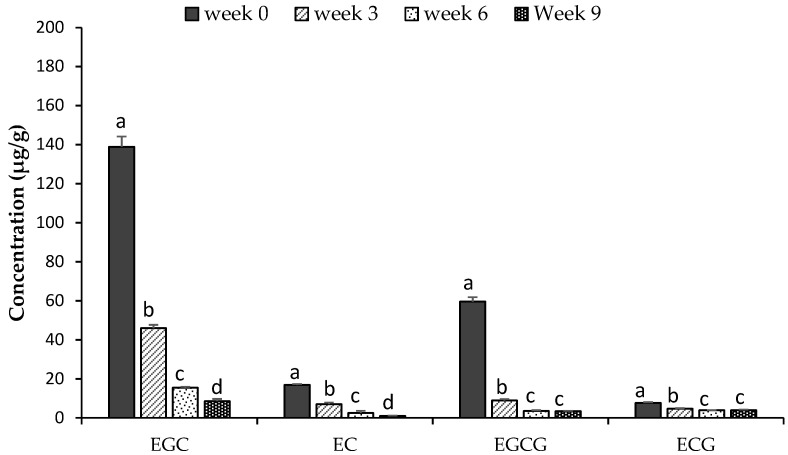
Catechins in DHA-rich oil supplemented with green tea extract at 1000 ppm and stored over 9 weeks at 30 °C. Values are presented as mean ± SD (n = 3). Different letters (a–d) for each catechin indicate a significant difference (*p* ≤ 0.05) during storage. EGC = epigallocatechin, EC = epicatechin, EGCG = epigallocatechin gallate, ECG = epicatechin gallate.

All the catechin compounds in GTE1000 significantly (*p* ≤ 0.05) declined during 6 weeks of storage and there was no significant change (*p* > 0.05) in the content of ECGC and ECG, thereafter (Figure 2). The depletion was higher for EGC and EGCG than for the other catechins during the first 3 weeks of storage with a percentage loss of 66.6% and 85.1%, respectively. This significant loss in catechins also corresponded with a lesser change in PV, which could imply the depletion of catechins was due to its antioxidant activity. Catechins are known to delay oxidation by inhibiting the formation of free radicals or blocking the propagation of the free radical chain reactions, as well as chelating metal ions [54,55]. Catechins, donate hydrogen from the phenolic-OH group, thus reducing free radicals. The resulting unpaired electron in the phenolic compound becomes delocalized within the ring structure giving a relatively unreactive free radical [56]. The depletion of catechins (% loss) was observed in the order EGCG > EGC > EC > ECG and this is similar to the order of antioxidant activity observed for catechins in mackerel muscle and oil-in-water emulsions [30,57].

### 3.5. Changes in Tocopherol Content of Oil during Storage

**Figure 3:**
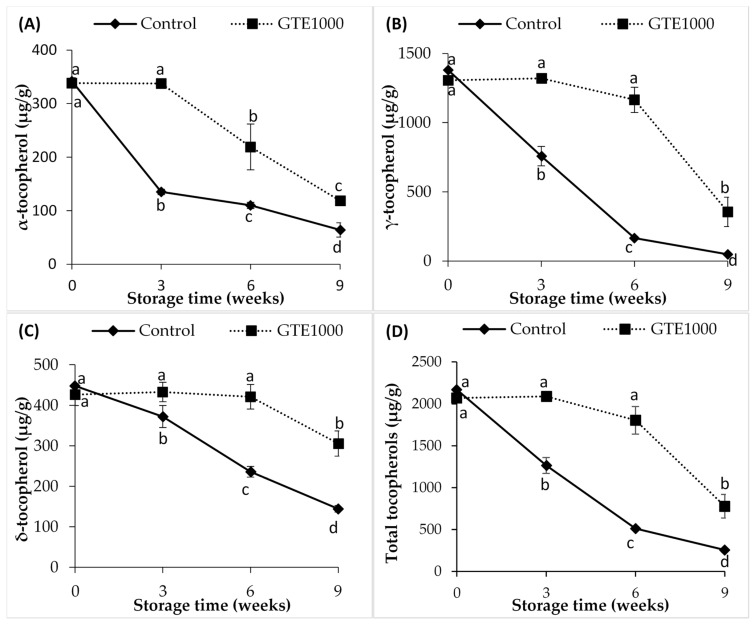
Tocopherol content of DHA-rich oil during 9 weeks of storage at 30 °C. (**A**) α-tocopherol; (**B**) γ-tocopherol; (**C**) δ-tocopherol; (**D**) Total tocopherols. Values are presented as mean ± SD (n = 3). Different letters (a–d) indicate a significant difference (*p* ≤ 0.05) during storage for each sample.

The α-tocopherol, γ-tocopherol and δ-tocopherol content of DHAO were 342.2 µg/g, 1380.9 µg/g and 448.3 µg/g, respectively. The oil was used without further purification and the effect of GTE-supplementation on the degradation of these tocopherols was evaluated (Figure 3A–D). A greater degradation rate of all tocopherol compounds was observed for the control sample. During the first 3 weeks of storage, α-tocopherol, γ-tocopherol and δ-tocopherol content of the control sample reduced by 60%, 45% and 17%, respectively and after 9 weeks, γ-tocopherol was the most degraded with a reduction of 96%, followed by α-tocopherol with 81% reduction and δ-tocopherol with a reduction of 67%. On the other hand, the α-tocopherol content of the GTE1000 samples remained stable during the first 3 weeks of storage while content of γ-tocopherol and δ-tocopherol remained stable for up to 6 weeks of storage. Losses in α-tocopherol, γ-tocopherol and δ-tocopherol of 65%, 72% and 28% were recorded for the GTE1000 sample after 9 weeks of storage. Thus, the incorporation of GTE in the oil reduced the depletion of the tocopherols with degradation levels ranked in the order of α-tocopherol > γ-tocopherol > δ-tocopherol. The differences in the rate of degradation of tocopherols are associated with their bond dissociation energy, which is in the order α < γ < δ and thus, α-tocopherol has a higher antioxidant activity followed by γ-tocopherol and then δ-tocopherol [58,59]. Accordingly, the rate of degradation was higher for α-tocopherol than for the γ-isomer in an oleic and linolenic acid-rich diacylglycerol oil, indicating a lesser stability for α-tocopherol [52]. The degradation of tocopherols in GTE1000 began after most of the catechins had been depleted. This result seems to corroborate the result of Jung et al. [60], which demonstrated that GTE was a better antioxidant than α-tocopherol for protection against singlet oxygen oxidation of linoleic acid. Prabsangob and Benjakul [61] also observed a delay in the frying-induced degradation of tocopherols in a soybean oil/tea seed oil blend when EGCG and EC were incorporated in the oil.

## 4. Conclusions

Supplementing DHAO with GTE significantly improved the oxidative stability of DHAO by delaying the formation of primary and secondary oxidation products as well as degradation of EPA, DHA and tocopherols. In contrast, the addition of α-tocopherol increased the formation of secondary oxidation products in the oil, therefore exhibiting a pro-oxidant tendency. The depletion of catechins matched the increase in PV, suggesting a predominant role of catechins in enhancing the DHAO oxidative stability. GTE could therefore serve as an alternative natural antioxidant to improve the shelf life of DHAO.

This study shows that hypersensitive oils with an extremely high content in PUFAs could be protected from oxidation by the incorporation of GTE. The oxidation of DHAO in this study was carried out at 30 °C and in open bottles, although refrigeration temperatures are recommended for the storage of such hypersensitive oils. It is therefore of interest to repeat this experiment at refrigeration temperatures and to evaluate the oxidation products obtained in the oil. In addition, the stabilizing impact of GTE should also be evaluated in complex food matrices supplemented with DHA-rich oils.

## Figures and Tables

**Table 1:** Fatty acid composition, tocopherol content and oxidative status of DHA-rich oil (DHAO).

	Characteristics	DHAO
Fatty acid composition (mg/g)	C16:1cis9	2.0 ± 0.13
	C18:1cis9	1.4 ± 0.07
	C20:4c5,c8,c11,c14	6.1 ± 0.17
	C20:5c5,c8,c11,c14,c17 (EPA)	149.3 ± 4.4
	C22:5c4,c7,c10,c13,c16	15.4 ± 0.5
	C22:5c7,c10,c13,c16,c19	54.7 ± 1.3
	C22:6c4,c7,c10,c13,c16,c19 (DHA)	626.7 ± 16.1
	c24:5c9,c12,c15,c18,c21	1.5 ± 0.1
	c24:6c6,c9,c12,c15,c18,c21	11.1 ± 0.28
	Total SFA	3.4 ± 0.84
	Total MUFA	4.9 ± 0.57
	Total PUFA	873 ± 22.84
Tocopherol (µg/g)	α	342.2 ± 3.6
	γ	1380.9 ± 7.1
	δ	448.3 ± 0.7
	Total	2171.4 ± 10.0
Oxidative status	Peroxide value (meq O_2_/kg)	2.0 ± 0.0
	*p*-anisidine value	5.2 ± 0.3

Values are means of three measurements ± SD. SFA = Saturated fatty acids, MUFA = Monounsaturated fatty acids, PUFA = polyunsaturated fatty acids.

**Table 2:** The EPA and DHA content (mg/g) of DHA-rich oil at baseline (week 0) and after 9 weeks of storage at 30 °C.

	EPA		DHA	
Treatment	Week 0	Week 9	Week 0	Week 9
Control	149.3 ± 4.4 ^aA^	136.8 ± 5.0b ^cB^	626.7 ± 16.1 ^aA^	582.8 ± 20.3 ^cdB^
Toco80	146.3 ± 1.9 ^aA^	134.6 ± 4.3 ^cB^	614.0 ± 7.5 ^aA^	570.8 ± 18.2 ^cdB^
Toco200	147.2 ± 0.9 ^aA^	129.8 ± 3.0 ^cB^	617.4 ± 3.4 ^aA^	550.5 ± 12.0 ^dB^
Toco500	141.6 ± 5.7 ^aA^	132.7 ± 8.6 ^cA^	593.9 ± 23.6 ^aA^	563.4 ± 35.2 ^cdA^
GTE160	138.7 ± 14.3 ^aA^	140.1 ± 1.94 ^abcA^	583.5 ± 58.3 ^aA^	599.89 ± 7.8 ^bcA^
GTE400	139.28 ± 7.3 ^aA^	146.5 ± 1.5 ^abA^	585.2 ± 28.2 ^aA^	628.2 ± 7.1 ^abA^
GTE1000	151.3 ± 8.8 ^aA^	149.57 ± 2.12 ^aA^	635.7 ± 36.9 ^aA^	644.0 ± 9.0 ^aA^

Values are mean ± SD (n = 3). Different superscript capital letters (^A or B^) indicate a significant difference (*p* ≤ 0.05) between week 0 and week 9 (row) for the same fatty acid; different superscript lower-case letters (^a–d^) within a column indicate significant differences (*p* ≤ 0.05) among samples at the specific storage time. Control = DHA-rich oil; Toco80, Toco200 and Toco500 = DHA-rich oil supplemented with 80 ppm, 200 ppm and 500 ppm of α-tocopherol, respectively; GTE160, GTE400, GTE1000 = DHA-rich oil supplemented with 160 ppm, 400 ppm and 1000 ppm of green tea extract, respectively.

**Table 3:** Catechin content (µg/g) of DHA-rich oil supplemented with green tea extract after 9 weeks of storage at 30 °C.

		EGC	EC	EGCG	ECG	Total
Control	T = 0 week	0.72 ^d^	0.07 ^d^	0.91 ^d^	0.31 ^d^	2.1 ^d^
GTE160	T = 0 week	22.7 ± 0.02 ^c^	2.8 ± 0.06 ^c^	9.7 ± 0.14 ^c^	3.6 ± 0.02 ^c^	39.4 ± 0.1 ^c^
	T = 9 weeks	10.3 ± 0.19 ^a^	1.3 ± 0.04 ^a^	4.4 ± 1.4 ^a^	3.9 ± 0.5 ^a^	22 ± 2.3 ^a^
	% loss	54.72	54.00	55.03	0.0	44.21
GTE400	T = 0 week	60.2 ± 1.5 ^b^	7.4 ± 0.20 ^b^	25.9 ± 0.56 ^b^	4.8 ± 0.06 ^b^	99.4 ± 2.1 ^b^
	T = 9 weeks	8.1 ±0.62 ^b^	1.1 ± 0.12 ^a^	3.6 ± 0.09 ^a^	4.1 ± 0.15 ^a^	19.1 ± 1.1 ^a^
	% loss	86.56	85.34	86.01	15.55	80.84
GTE1000	T = 0 week	138.9 ± 5.3 ^a^	16.8 ± 0.5 ^a^	59.5 ± 2.3 ^a^	7.6 ± 0.39 ^a^	224.8 ± 7.5 ^ab^
	T = 9 weeks	9.5 ± 1.22 ^ab^	1.2 ± 0.49 ^a^	3.02 ± 0.2 ^a^	4.3 ± 0.16 ^a^	20.1 ± 2.14 ^a^
	% loss	93.19	92.77	94.93	43.54	91.05

% loss = (amount at T = 0–amount at T = 9 weeks) × 100/amount at T = 0 week. Values are presented as mean ± SD (n = 3). Different superscript letters (^a^–^d^) within a column indicate significant differences (*p* ≤ 0.05) among samples at the same storage time. EGC = epigallocatechin, EC = epicatechin, EGCG = epigallocatechin gallate, ECG = epicatechin gallate. GTE160, GTE400, GTE1000 = DHA-rich oil supplemented with 160 ppm, 400 ppm and 1000 ppm of green tea extract, respectively.

## Data Availability

Data is contained within the article.
